# Ilizarov methodology for infected non union of the Tibia: Classic circular transfixion wire assembly vs. hybrid assembly

**DOI:** 10.4103/0019-5413.33682

**Published:** 2007

**Authors:** Ranjit Kr. Baruah

**Affiliations:** Department of Orthopedics, Assam Medical College, Dibrugarh - 786 002, Assam, India

**Keywords:** Hybrid assembly, Ilizarov, infected nonunion, conventional (classic circular transfixion wire) Ilizarov assembly

## Abstract

**Introduction:**

Conventional wire fixation of Ilizarov rings often fails to provide 90-90 configuration because of vital structures, which is essential for optimum stability. Hybrid assembly with half pins is an alternative. The aim of this study is to compare the results of Hybrid assembly with that of conventional classic circular transfixion wire Ilizarov assembly in 50 cases of infected nonunion of tibia between 1994 and 2003.

**Materials and Methods:**

This study includes two groups with 25 patients in each group: Group (A) conventional Ilizarov assembly and Group (B) hybrid Ilizarov assembly. Thirty-five cases developed infected nonunion following road traffic accidents while others after fall (6) bullet injury (4), infected osteosynthesis (3) and assault (2). There were 45 males and five females with mean age (18 to 56 years). All active cases (n=28) were treated by debridement including removal of implants in infected osteosynthesis. Twenty out of 22 cases in the quiescent group (non draining for last three consecutive months) were treated without open debridement; only two cases required open debridement for various reasons. All the cases were finally treated as atrophic aseptic nonunion with bone defect and were classified according to ASAMI.

Type B1: length of the limb maintained with bone gap (14 cases in both Group A and B) and Type B3: combined shortening with defect (five and seven cases in Group A and B respectively), were treated by bifocal osteosynthesis. Only one case in the B3 group was treated by trifocal osteosynthesis to shorten the time. Type B2: segments in contact with limb shortening (total nine cases; five and four cases in Group A and B respectively) with shortening up to 2 cm (total five cases) were treated with monofocal osteosynthesis while shortening up to 5 cm and beyond (total four cases) were treated with bifocal osteosynthesis.

**Results:**

The cases were followed up for two to six years and the results were evaluated by Paley criteria of bony results (union, infection, deformity and leg-length discrepancy) and Functional Results (significant limp, equinus rigidity of the ankle, soft-tissue dystrophy, pain and inactivity). In both the groups, 24 cases out of 25, had excellent to good bony result with Group B having twice more excellent result than Group A. Functional results were found to be similar in both the groups. Although persistence of infection and Grade III pin tract infection (PTI) were slightly higher in Group B, complications like delayed consolidation of regenerate, refracture, deformity and aneurysm of vessel were less in this group.

**Discussion and Conclusion:**

Ilizarov methodology produced a satisfactory result in infected nonunion of the tibia. Hybrid assembly was a fruitful advancement in the Ilizarov armamentarium. The results were comparable to Conventional assembly in terms of docking site problems, corticotomy site problems, PTIs and other problems.

Goals of treatment in infected nonunion are to obtain solid bony union, eradication of infection with maximum functional use of the extremity.^1^

Different modalities of management like extensive debridement and local soft tissue rotational flaps, packing the defect with antibiotic impregnated beads, Papineau type cancellous bone grafting, tibio-fibular synostosis, cancellous allograft in fibrin sealant mixed with antibiotics and or free micro vascular soft tissue and bone transplants etc are described to treat infected nonunions. The essence of these operations is to create an infection-free environment and then to proceed for stabilization for the stimulation of healing potential. They are usually staged requiring one or more reconstruction procedures and thus prolonging the recovery period. Moreover they have variable rates of success and failures and they are unable to reestablish extremity length and correct deformity. They fail to give early functional rehabilitation during treatment. On the other hand Ilizarov has been found to show encouraging results in infected nonunion of tibia as it can not only offer a one-stage solution to varieties of problems associated with infected nonunion namely, infection, shortening and deformity, but also allow early weight bearing during treatment. It produces regenerate without bone graft.^2^–^9^

Hybrid assembly is an advancement of the original Ilizarov apparatus introduced by the Lecco group in Italy in 1986, where half pins were used in certain areas in place of wires.^10^,^11^

An all-wire frame has the following disadvantages:

1. Muscle and tendon transfixation leads to pain and contracture of adjacent joint.
2. Chances of neurovascular impalement
3. Olive wires are more painful and their removal is difficult
4. 90-90 placement of wires is not always possible particularly in sites like the proximal humerus and femur and distal femur compromising the stability of assembly.

Green (1991) started using half pins with the above facts in mind.^12^ He compared the results with that of conventional Ilizarov assembly with encouraging results. He was of the opinion that substitution of wires with half pins reduced pin site sepsis, reduced patient discomfort, lessened the need for analgesics and shortened the overall time of external fixation by enhancing ossification and maturation of the regenerate new bone.^13^

We have treated infected nonunion of the tibia by the Ilizarov method. We have used both conventional and Hybrid assembly to treat such cases. The aim of this study is to compare the results of Hybrid assembly being used in infected nonunion of the tibia with conventional Ilizarov, specially in terms of rate of union along with complications like pin tract infection, hyporegenerate, nonunion at corticotomy site, nonunion, refracture etc. in both the groups.

## MATERIALS AND METHODS

Fifty patients of infected nonunion of the tibia, treated during 1994 to 2003 were included in the present analysis. It is a retrospective study. We used conventional Ilizarov assembly in the first 25 cases which were included in Group A, while the next 25 cases treated by hybrid assembly were included in Group B.

Group A (n = 25) patients treated with conventional Ilizarov. The age ranged from 18 to 50 years with 22 males and three females. Infected nonunion developed following open fracture in 22 cases and infected osteosynthesis in three cases. The causes of open fractures were road traffic accidents (RTA) in 16 cases, fall in four cases and bullet injury in two cases. There were 20 cases of Type 3 and two cases of Type 2 open fractures (Gustilo type).

Group B (n = 25) patients treated with hybrid Ilizarov assembly. The age ranged from 18 to 56 years with 23 males and two females. Infected nonunion developed following open fracture in all cases and the causes of open fractures were RTA in 19 cases, fall in two cases, assault in two cases and bullet injuries in two cases. There were 13 cases of Type 3 and 12 cases of Type 2 open fractures (Gustilo type).

All the open fractures (n=47), except two cases, were treated initially by debridement and stabilization with unilateral tubular external fixator. Plastic reconstructions were done to cover the exposed bones in 16 cases; cross leg flap in two cases, local rotational flaps in five cases and split-skin graft in the rest of the cases. Two cases of open fracture in Group B were initially treated by primary Ilizarov without excision of bone. Persistence of infection in them required further debridement and corticotomy; only three cases had infected nonunion following failed infected osteosynthesis.

With slight modification of the classification for infected nonunion as advocated by Rosen,^14^ we divided our patients into two categories, Active and Quiescent depending on the status of infection to postulate the treatment protocol. The active group included both the actively draining and active non-draining cases for less than three months of Rosen. Cases without drainage or symptoms for three months or more were termed as quiescent. Debridement was done in all active cases to eradicate infection; active draining cases required extensive debridement whereas active non-draining cases required relatively less extensive debridement. Cases in the quiescent group did not require debridement except in a few situations as described in Methods below. They were finally labeled as atrophic nonunion with gap and were classified into three types according to Association for the Study and Application of Ilizarov's Method (ASAMI);^15^ B1: length of the limb maintained with bone gap, B2: segments in contact with limb shortening, B3: combined shortening with defect.

In our series, Group A had 17 active and eight quiescent cases while Group B had 11 active and 14 quiescent cases. Twenty cases had diaphyseal and five cases had metaphyseal involvement in Group A whereas Group B had 17 with diaphyseal and eight with metaphyseal involvement. The gap ranged from 2-9 cm in both the groups with mean gap varying (4.36 cm and 2.64 cm in Group A and Group B respectively).

All active cases (total 28 cases) were treated by debridement including removal of implants in infected osteosynthesis. The majority of cases (20 out of 22 cases) in the quiescent group were treated without open debridement. All the cases were finally treated as atrophic aseptic nonunion with bone defect according to ASAMI. B1 (14 cases in both Group A and B) and B3 (five and seven cases in Group A and B respectively) cases were treated by bifocal osteosynthesis. Only one case in the B3 group was treated by trifocal osteosynthesis to shorten the time. B2 cases (total nine cases; five and four cases in Group A and B respectively), with shortening up to 2 cm (total five cases) were treated with monofocal osteosynthesis while shortening up to 5 cm and beyond (total four cases) were treated with bifocal osteosynthesis.

Only two cases in the quiescent group underwent open debridement. In a case of 12 years old nonunion following segmental open fracture, with dense cortical bone at the fracture site, open debridement was done to excise dense bony ends, resulting in 3 cm of gap for which Bifocal osteosynthesis was done. In another case, who had many loose separated comminuted fragments without probably any soft tissue attachments and hence doubtful vascularity, also underwent open debridement.

In Group A 1.8 mm K-wires were used according to Wire protocol and Stainless steel Schanz screws were used in all the cases in Group B. The size of the Schanz screws was 4.5 mm with short threads so that the distal cortex was only engaged by the threaded part with the 5 mm shaft engaging the near cortex producing radial preload.

Meticulous pin insertion technique, as mentioned below, was followed to get optimum results:

1. Cruciate skin incision
2. Periosteum raised to either side
3. Good quality SS drill bit was used with hand drill in the technique of “stop and go” with continuous saline irrigation
4. Drill bit was taken out from time to time to clean it of bone debris
5. Saline blast of the drill hole was done after drilling to wash the bone debris from inside the hole
6. Short-threaded, measured Schanz screw of appropriate length was introduced to get a radial preload. It was introduced in splaying manner and obliquely to fracture site to negate the cantilever action.

Regarding placement of the Schanz screws, the following protocol was followed:

1. They were put in the diaphysis only
2. Metaphysis was always fixed with conventional wires
3. Out of two wires being put in a ring in all wire frame, only one wire was substituted with one screw; the screw was put at right angle to the wire (T-fashion). This made at least one Schanz screw being put in each fragment.

Postoperative pin tract care was meticulously followed. Patients were encouraged to bear weight as early as possible. Patients were discharged only when docking was achieved and limb length was attained. No soft tissue reconstruction and bone grafting were done in any of the cases. Regular follow-up of cases were done in the OPD and assessed for pin tract infection (PTI), loosening of components, healing of soft tissue and fractures and any other complications.

## RESULTS

We followed up the cases for two to six years and evaluated the results. In all cases, four-ring assembly was used. In three cases, foot assembly with ankle joint spanning was incorporated to stabilize a small distal fragment. The Gap ranged from 2-9 cm in both the groups. The mean gap in Group A was 4.36 cm and in Group B was 2.64 cm. Mean lengthening achieved was 3.7 cm in B2 cases and 3 cm in B3 cases. Mean hospital stay was 14.5 weeks and 12 weeks in Group A and B respectively. Bone grafting was not done in any of the cases. Change of wire was required in one case in Group A for PTI not responding to rest and antibiotics, showing increasingly painful site. In another case that developed a false aneurysm of the lateral peroneal artery, the offending wire was removed while the vessel was ligated to control hemorrhage. Fixator was retained for a longer period of time in Group A (mean 195.44 days and 169.5 days in Group A and B respectively). Union time was more in Group A while angulations of the regenerate were found to be similar in both the groups [Table 1].

**Table 1:** Showing fixator duration, union and angulations

Group	Fixator duration days (mean)	Union weeks (mean)	Angulation degrees (mean)
A	100-445 (195.44)	17-78 (29.25)	2-16.5 (7.24)
B	110-286 (169.5)	16-34 (23.62)	4-16 (6.75)

One case in Group A underwent amputation through leg as there was failure to union with inactive, stiff distal leg and foot. Although there was union, one case in Group A and two cases in Group B had some infection at the original nonunion site, occasionally discharging tiny bony pieces which eventually healed up in the latest follow-up.

The final results were evaluated by Paley criteria of bony results and functional results.^8^ For bone results, four criteria were evaluated: union, infection, deformity and leg-length discrepancy. An excellent bone result was one with union, no infection, deformity of less than 7° and leg-length discrepancy of less than 2.5 cm in the tibia. A good result was union plus any two of the others. A fair result was union plus one of the others. A poor result was nonunion or refracture or none of the others. According to these criteria, there were eight and 16 excellent results and 16 and eight good results in Group A and B respectively. There was one poor result each in both the groups and there were no fair results.

The functional results were based on five criteria; significant limp, equinus rigidity of the ankle, soft-tissue dystrophy (skin hypersensitivity, insensitivity of sole or decubitus), pain and inactivity (unemployment because of the leg injury or inability to return to daily activities because of the leg injury). An excellent result was an active individual with none of the other four criteria; a good result was an active individual with one or two of the other four criteria; and a fair result was an active individual with three or four of the other criteria or an amputation. An inactive individual was considered a poor result regardless of the other criteria. According to these criteria, functional results were found to be similar in both the groups. There were 23 and 22 cases of excellent to good results in Group A [Figure 1 A–G] and B [Figure 2 A–E] respectively. There were one each fair and poor results in each group.

**Figure 1:**
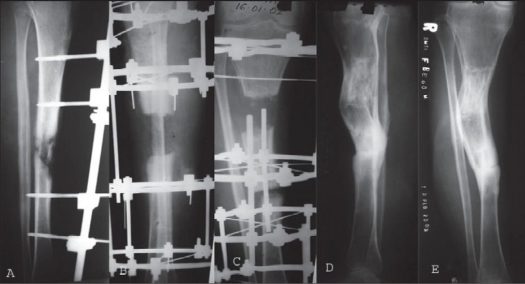
Conventional Ilizarov assembly. A) Open tibial fracture treated initially with debridement and unilateral external fixator. B) Second debridement and conventional Ilizarov frame application. C) Follow up X-ray depicting internal bone transport. D and E) X-ray post frame removal and fracture consolidation

**Figure 1 F and G:**
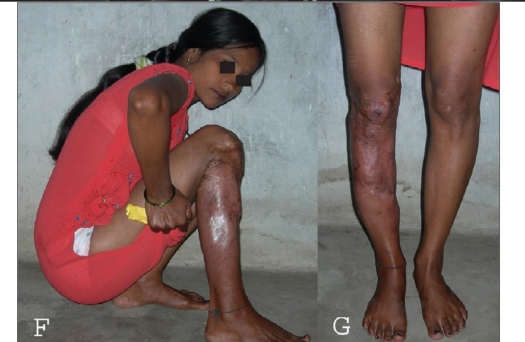
Final functional result of the same patient

**Figure 2:**
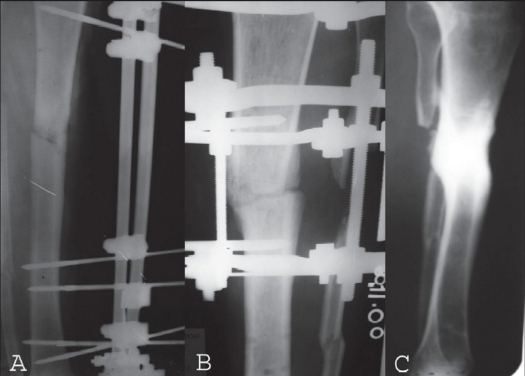
Hybrid assembly. A) Open tibial fracture treated initially with debridement and unilateral external fixator. B) X-ray depicting Hybrid assembly and early fracture union. C) X-ray post frame removal showing fracture consolidation

**Figure 2 D and E:**
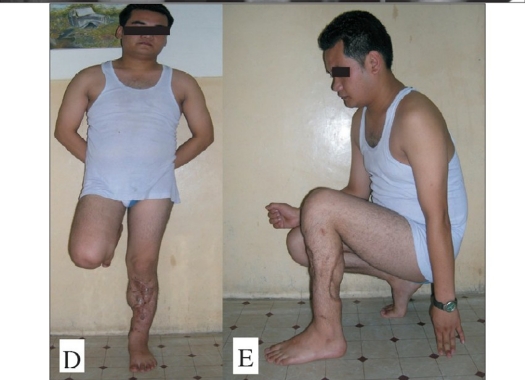
Functional outcome of the same patient.

PTI were classified into three groups according to Paley;^16^ Grade 1 soft tissue inflammation, Group 2 soft tissue infection and Grade 3 bone infection. Complications were further highlighted in the following way [Table 2].

**Table 2:** Showing complications in both the groups

	Group A [=n]	Group B [=n]
1. Docking site problems		
a) Persistence of infection	01	02
b) Delayed consolidation	02	01
c) Interposition of soft tissue	04	02
d) Nonunion	01	01
e) Refracture	02	00
2. Corticotomy site problem		
a) Hyporegenerate	02	02
b) Early consolidation	01	00
c) Delayed consolidation	02	02
d) Infection	01	00
e) Nonunion	01	01
3. Gr.III PTI	00	01
4. Deformity equines	02	00
5. Others aneurysm of vessel	01	00

## DISCUSSION

All the available definitions of nonunion and delayed union, including one by Food and Drug Administration (FDA) of USA and another by Muller, are inconsistent, subjective, ambiguous and arbitrary and therefore do not have any objective criteria. For example, several months of observation should not be required to declare a tibia shaft fracture with 10 cm of segmental bone loss a nonunion. We therefore follow the definition contemplated by Brinker. It defines nonunion as a fracture that, according to the treating physician, has no possibility of healing without further intervention.^1^ Infected nonunion is a post-traumatic bony wound and not equivalent to hematogenous osteomyelitis. Out of several parameters that help to identify infected nonunion of a tibia fracture as cited by Toh and Jupiter, exposed bone that has been devoid of vascularized periosteal coverage for more than six weeks and purulent drainage were considered to make a diagnosis of infected nonunion.^17^

Since its first incorporation in the assembly, the half pin has increasingly being used as advocated by Green, Catagni, Paley with uniformly good results. The half pin eliminates the disadvantages of an all-wire frame it is user-friendly and less time-consuming. Patient tolerability is better and that helps in early physiotherapy. According to Green, Paley etc., half pin doesn't reduce the overall elasticity; it rather improves the stability of the system. On the other hand, Ilizarov himself never related his success to the elasticity of the device but always stressed the importance of assembly stability.^18^

The Lecco group has classified the Ilizarov assembly into three types:

1. Conventional all wires
2. Hybrid traditional [HT] minimum half pins
3. Hybrid advance [HA] maximum half pins

We have been using HT assembly in our cases in various situations. We are using stainless steel half pins in all cases. Although SS pins seem to develop more PTIs [Green *et al.*], this can be taken care of by meticulous pin insertion technique as mentioned earlier as well as strict postoperative protocol.

While comparing the results, we found that docking site problems like persistence of infection were slightly higher in Group B [8% as compared to 4% in Group A] as well as Gr. III PTI [4% as compared to 0% in group A]. The incidences of delayed consolidation of fracture site and refracture were far less in Group B [8% and 8% respectively in Group A as compared to 4% and 0% respectively in Group B]. But the difference was not statistically significant (*P* > 0.05) in all the results as mentioned above. However, incidences of nonunion at fracture site were the same [4%] in both groups. In case of corticotomy site problems, incidences of hyporegenerate and delayed consolidation and nonunion were the same in both the groups.

On the other hand, deformity in the form of equinus was commonly seen in the Conventional group [8%] which was not seen at all in the Hybrid group. The two cases that developed equines were B3 cases requiring correction of limb length discrepancy along with the gap. Impalement of muscles leading to difficulty in physiotherapy could be the cause of such a deformity in this group. Regarding Pin site problems, Gr. I and II PTIs of Paley (i.e., Soft tissue Inflammation and Soft tissue infection respectively) were almost same in both the groups; but Gr. III (bone infection) was seen in 4% of cases in Gr. B. Aneurysm of the lateral peroneal artery developed in one case in Group A.

Contrary to observations made by Calhoun and co-workers,^19^ substitution of one wire with one Schanz screw was sufficient to maintain the stability as we put only one Schanz screw and one wire in the diaphysis in our cases.

## CONCLUSION

The results of Hybrid assembly in infected nonunion of the tibia are comparable to Conventional assembly in terms of docking site problems, corticotomy site problems, PTIs and other problems. It is user-friendly for an average surgeon. It is therefore safe to conclude that Hybrid assembly is a fruitful advancement in the Ilizarov armamentarium.
